# “Our needs, our priorities, listen to us!” recommendations for improving HIV prevention and the cascade of care from people living with HIV in Manitoba, Canada: a qualitative study

**DOI:** 10.1186/s12913-025-12645-5

**Published:** 2025-04-23

**Authors:** Enrique Villacis-Alvarez, Margaret Haworth-Brockman, Katharina Maier, Cheryl Sobie, Heather Pashe, Joel Baliddawa, Nikki Daniels, Rebecca Murdock, Robert Russell, Clara Dan, Freda Woodhouse, Susie Cusson, Lisa Patrick, Marj Schenkels, Michael Payne, Ken Kasper, Lauren J. MacKenzie, Laurie Ireland, Kimberly Templeton, Yoav Keynan, Zulma Vanessa Rueda

**Affiliations:** 1 Department of Medical Microbiology and Infectious Diseases, University of Manitoba Rady Faculty of Health Sciences, Winnipeg, MB R3E 0J9 Canada; 2 Department of Community Health Sciences, Rady Faculty of Health Sciences, University of Manitoba, Winnipeg, MB R3E 0J9 Canada; 3 National Collaborating Centre for Infectious Diseases, Rady Faculty of Health Sciences, University of Manitoba, Winnipeg, MB R3E 0T5 Canada; 4 Department of Criminal Justice, The University of Winnipeg, 515 Portage Ave, Winnipeg, MB Canada; 5 Peer Research Team, Alltogether4IDEAS, Winnipeg, MB R3E 0J9 Canada; 6 The Manitoba HIV Program, 705 Broadway Ave, Winnipeg, MB R3G 0X2 Canada; 7 Nine Circles Community Health Centre, 705 Broadway Ave, Winnipeg, MB R3G 0X2 Canada; 8 Department of Internal Medicine, University of Manitoba Rady Faculty of Health Sciences, Winnipeg, MB R3E 0J9 Canada; 9 Department of Family Medicine, University of Manitoba Rady Faculty of Health Sciences, Winnipeg, MB R3E 0J9 Canada

**Keywords:** HIV, Mental health, HIV care implementation, Community-based research, Qualitative research

## Abstract

**Background:**

The Canadian province of Manitoba has reported a 52% increase in HIV diagnoses during the past 5 years. Females are disproportionately affected by HIV and multiple intersecting health and social challenges, including houselessness, injection drug use, sexually transmitted and blood borne infections, and mental health conditions. Program and service development often ignore people’s complex lived experiences. Our aim was to describe recommendations made by people living with HIV (PLHIV) to inform a person-centred HIV cascade of care valuing the needs and ideas from PLHIV.

**Methods:**

This qualitative study was conducted between October 2022 and May 2023. Thirty-two women, men, and gender-diverse participants completed a semi-structured interview. Interviews were recorded, transcribed, and analyzed using NVivo 12, deploying thematic analysis to understand major themes related to recommendations to care. This manuscript focuses on questions related to recommendations for the HIV cascade of care.

**Results:**

Recommendations fell within two major themes: ‘Meeting people where they are at’ and an HIV educational strategy. The first theme included three main categories to make HIV services more accessible. (1) psychological (social programming, peer support during diagnosis, increased mental health services), (2) biomedical (HIV outreach, HIV services outside 9 –5 h, specialized care outside metropolitan areas, universal coverage for HIV medicines), and (3) social (transportation support, emergency housing, financial support) supports. The HIV educational strategy included five major categories: (1) physical posters and billboards in highly transited areas; (2) community meetings with peer-led education; (3) comprehensive sex education in schools; (4) training primary healthcare providers on stigma and discrimination; (5) and social media campaigns to reach younger audiences. We report on gender differences for recommendations where they arose. The themes described by PLHIV suggest a need to implement HIV care delivery models that will connect and maintain people in HIV care in Manitoba.

**Conclusions:**

This study provides practical and person-centred strategies that could bridge the barriers PLHIV face when accessing and remaining in HIV care and expanding education and prevention about HIV in Manitoba.

## Background

For two consecutive years, the province of Manitoba, Canada, has reported its highest ever increase in HIV diagnoses [1, 2]. People newly diagnosed with HIV often experience intersecting health and mental health disparities and social-structural marginalization [1, 2]. In Manitoba, the ratio female: male is similar (102 females and 93 males diagnosed with HIV in 2022) and females newly diagnosed with HIV have higher proportions of houselessness, injection drug use, co-infections of sexually transmitted and blood-borne infections (STBBIs), and mental health conditions [1–4]. This highlights the need for holistic and gender-based recommendations for HIV care models, grounded in people’s lived and gendered experiences. Similar to other places, people living with HIV (PLHIV) in Manitoba often lack proper access to (mental) health, addictions services, and accessible HIV care, such as HIV outreach and social programs; for people experiencing houselessness and substance use dependence, barriers to care are often exacerbated [5]. Moreover, PLHIV report experiencing stigma and discrimination by primary healthcare providers and social networks, creating additional barriers to care [5]. Addressing these barriers in an effort to connect to and maintain PLHIV’s care is crucial to improve health outcomes and prevent transmission.

Communities and advocates in HIV care have actively sought to participate in policy and decision-making since the start of the HIV epidemic [6–9]. Many activists emphasize the need for greater involvement of people living with HIV principles which emphasize PLHIV’s agency and right to participate in decision-making processes related to their health by providing opportunities for growth, connection, empowerment, and decision-making [9]. Health providers and organizations also benefit from for greater involvement of people living with HIV as they gain first-hand knowledge and insights from their clients’ experiences with their services [9, 10]. However, challenges such as low skills level to engage in projects and funding constraints to meaningfully involve PLHIV can limit PLHIV’s engagement in HIV programs and system designs [9]. For example, researchers in Ontario found challenges, particularly among underserved groups, of incorporating these principles into the practice of HIV organizations. Some of these challenges related to the contextual setting such as PLHIV facing stigma and discrimination by disclosing their HIV status and engaging with HIV organizations. Other challenges found by the researchers included the limiting circumstances PLHIV can actively engage with these organizations. For instance, it was found that HIV organizations offer volunteer or part-time positions with very specific skill set requirements that not many PLHIV could apply to which leaves many disconnected from engaging in this work [10]. Their findings emphasize that even within HIV specific environments, PLHIV’s experiences are not been prioritized so that they could have an input into changes to HIV services. With these findings in mind, our objective was to listen to and outline the recommendations made by PLHIV to improve HIV prevention and the HIV cascade of care to create a more person-centred and accessible model of care.

## Methods

We conducted 32 in-depth semi-structured interviews with PLHIV in Manitoba between October 2022 and May 2023. The goal was to understand PLHIV’s recommendations to make HIV care more accessible in Manitoba considering the rising numbers of new HIV diagnoses. Study Setting.

Located in central Canada, Manitoba has the 5th highest population of all Canadian provinces [11]. Most of the 1.4 million people in the province live in the cities of Winnipeg (~ 900,000) and Brandon (~ 55,000). This study was conducted in the three clinic sites of the Manitoba HIV Program, two located in Winnipeg and one in Brandon. The Winnipeg Regional Health Authority accounts for the region with the majority of new HIV diagnoses in Manitoba at 65.7% in 2021 [2]. Two of these clinics provide many resources beyond biomedical services such as harm reduction resources, financial resources, showers, and washing machines. Most clients from these clinics are already connected to care, though people who are in and out of care also access their services.

### Participants

Participants had to be at least 18 years old and reside in Manitoba. A Research Advisory Committee, Peer Research Team, Indigenous Cultural Advisor, HIV service providers, and community stakeholders informed the inception, development, and data analysis for this study to ensure the research remains grounded in PLHIV’s lived experiences [12]. We used purposive sampling to recruit women, men, and gender-diverse people from different backgrounds (e.g., ages, cultural background, languages spoken) and with various experiences in HIV care (e.g., participants who have been in HIV care continuously since their diagnosis, participants who have been in-and-out of HIV care). Fifty-four people expressed interest in participating in this study. The final sample was 32 participants after 21 did not meet the inclusion criteria, and one person withdrew after completing the initial consent form for personal reasons.

### Data collection

Recruitment and data collection processes were carried out by the EVA, peer co-researchers, and the Indigenous Cultural Advisor. Detailed information on the extensive community consultations for the data collection process and tools can be found elsewhere [5, 12]. The interview guide, for example, was co-developed with people with lived experience of substance use, houselessness, and interpersonal violence, as well as HIV and is fully accessible in the published protocol [12]. Questions related to recommendations included “What changes do you think would make it easier for you (and other people living with HIV) to access care?” and “What do you think is the best way [how do you want] to learn about prevention and treatment of STBBI’s and harm reduction practices?” After obtaining participants’ verbal or written consent, participants completed an in-depth semi-structured interview [12]. Participants received $50 CAD for their time, and reimbursement for any transportation and childcare costs to enable their participation. Interviews were audio recorded, transcribed using Otter.ai, reviewed for accuracy by EVA, and coded using NVivo 12. This manuscript focuses primarily on the interview data related to recommendations to HIV prevention and HIV cascade of care.

### Data analysis

We used thematic analysis, originally developed by Braun and Clarke [13], which facilitates deeper connection with the data, and allows researchers to recognize their subjectivity in the development of patterns [14]. Qualitative data analysis started with open coding by EVA, who led the data collection in HIV clinics, to identify all potential recommendations to HIV care in participants’ conversations. Twenty-seven changes and recommendations were initially created from the data. The larger research team reviewed the initial codes to group recommendations under larger themes. To decide on different pattern and themes among the recommendations, the research team discussed which specific areas of HIV care the recommendations fell under. Understanding that HIV care is a complex continuum, we wanted to agree upon and define themes that would be most helpful for making changes in HIV care in Manitoba. We found two major themes for recommendations: “Meeting people where they are at”, and an HIV educational strategy. Meeting people where they are at includes three categories (psychological supports, biomedical supports, social supports) and the HIV educational strategy includes five categories (printed posters and billboards, community meetings, sex education in schools, primary healthcare providers, social media campaigns). We looked for gender differences across categories and report on them as they appeared.

## Results

Table 1 reports the sociodemographic information of participants interviewed in this study.

**Table 1:** Sociodemographic information of people living with HIV in Manitoba interviewed in this study

**Variable (N)**	**Frequency** **n (%)**
Age in years (*N* = 32) Mean (Range)	44·03 years (24– 63)
Gender Identity (N=32)	
Woman	10 (31·3)
Man	18 (56·3)
Trans Woman	0
Trans Man	0
Non-Binary	1 (3·1)
Two-Spirit^a^	2 (6·3)
Other	1 (3·1)
Prefer not to say	0
Sex (*N*=32)	
Male	21 (65·6)
Female	10 (31·3)
Intersex	0
Prefer not to say	1 (3·1)
Sexual Orientation (*N*=32)	
Lesbian	0
Gay	8 (25)
Bisexual	6 (18·8)
Asexual	0
Heterosexual	15 (46·9)
Pansexual	0
Other	2 (6·3)
Prefer not to say	1 (3·1)
Cultural Background (*N*=32)	
Indigenous– First Nations	15 (46·9)
Indigenous– Métis	4 (12·5)
White/European	4 (12·5)
Southeast Asian	4 (12·5)
Other	5 (15·6)
Marital Status (*N*=27)^b^	
Single	18 (56·3)
Married	0
Divorced	2 (6·3)
Common Law	7 (21·9)
Widowed	0
Other	0
Highest Level of Education (*N*=30)^b^	
K-12	21 (65·6)
Certificate, Diploma, vocational course from an educational institution	6 (18·8)
Bachelor's Degree	3 (9·4)
Master’s Degree	0
Doctorate	0
Other	0
Income (*N*=31)^b^	
<10,000 CAD/Year	12 (37·5)
10,000– 19,999 CAD /Year	8 (25)
20,000 - 29,999 CAD /Year	2 (6·3)
30,000 - 39,999 CAD /Year	3 (9·4)
40,000 - 49,999 CAD /Year	3 (9·4)
>50,000 CAD /Year	1 (3·1)
Prefer not to say	2 (6·3)
Housing Situation^c^	
Living Alone (*N*=32)	10 (31·25)
Living with Partner (*N*=32)	6 (18·75)
Living with Children (*N*=32)	3 (9·38)
Living with Roommates (*N*=32)	5 (15·63)
Living with Extended Family (*N*=31)^b^	8 (25)
Experiencing Housing Instability (insecure housing, shelter, transitional housing, houseless) (N=32)	14 (43·75)

^a^Two-Sprit refers to an umbrella term used by Indigenous people to capture the experiences of people who do not fit within the male and female binary [15]. The Canadian Institutes of Health Research defines Two-Spirit as “Community organizing tool for Indigenous Peoples of Turtle Island [Canada] who embody diverse sexualities, gender identities, roles and/or expressions”

^b^Not all participants wanted to reveal identifiable information

^c^Not mutually exclusive categories

**Fig. 1:**
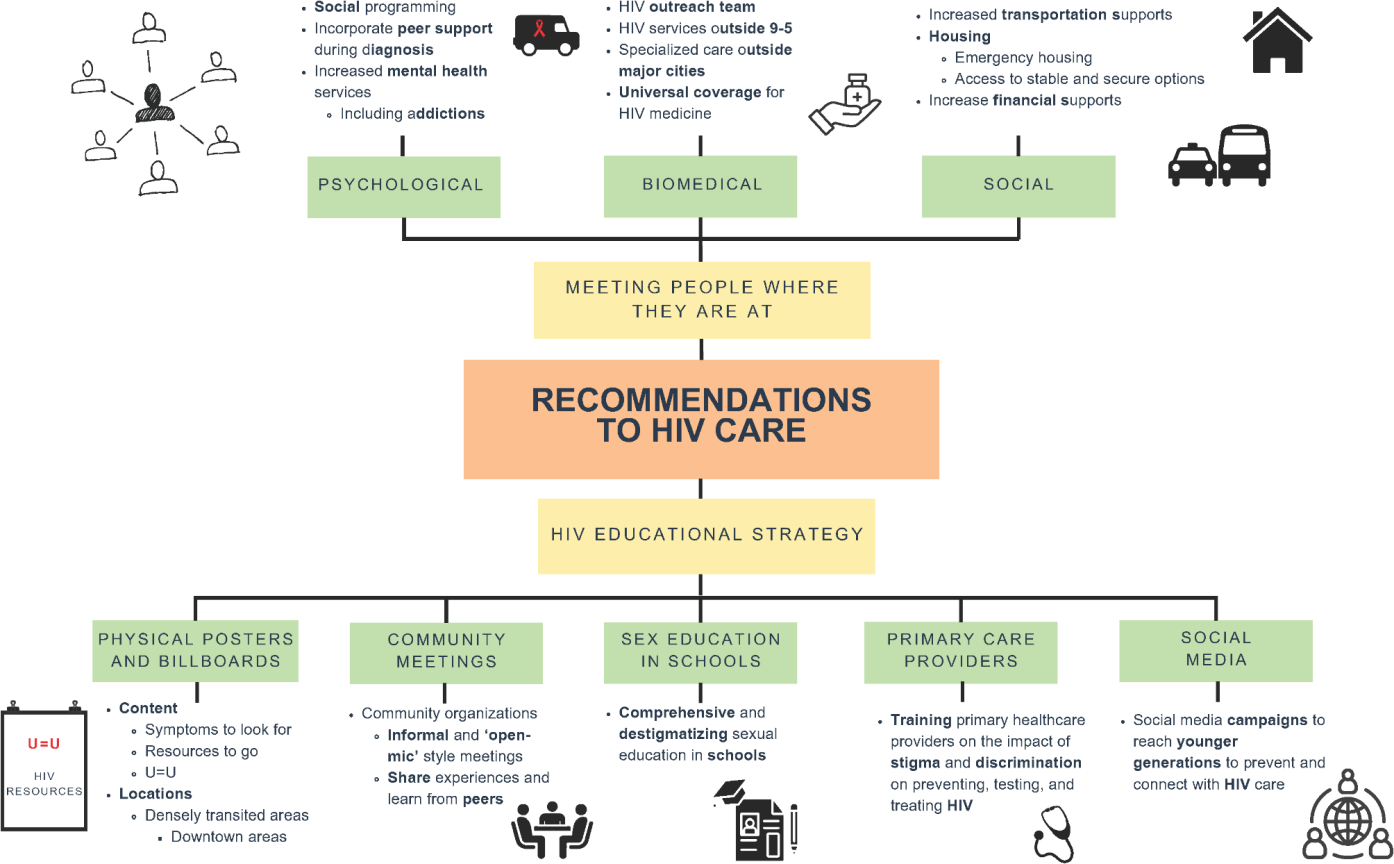
Mind map of recommendations to HIV cascade of care from people living with HIV in Manitoba

Qualitative findings from 32 participants are presented below. Figure 1 portrays the themes, categories, and subcategories through a mind map. Women, men, and gender-diverse participants often brought the same kinds of recommendations forward; however some findings differed by gender, which we detail below.

## Meeting people where they are at

Many participates talked about the challenges of accessing ‘traditional’ HIV care models (e.g., fixed appointments between 9 am-5 pm). Thus, many participants suggested making HIV care more “flexible” to enhance opportunities for care. According to participants, a “flexible” HIV care model includes proactive outreach and treatment (i.e., actively going to people who need HIV care rather than waiting for them to come to the HIV clinics on their own) while wrapping people around psychosocial supports. Findings revealed three main categories of support that are needed for accessible and optimal care: (1) psychological (2), biomedical, and (3) social.

### Psychological supports

#### Mental health services

Participants emphasized the need to expand mental health services as those currently available are insufficient and fail to meet PLHIV’s needs. According to participants, PLHIV routinely struggle with trauma, mental health conditions, or substance use dependence. They explained that dealing with mental health conditions and/or substance use can make it difficult to seek out and attend to HIV care. Providing swift and accessible linkage to mental health professionals and addiction services would better enable people to seek out and stay involved in HIV care.

*I went to the hospital because I wanted to quit drugs because my drug addiction was taking a toll on my mental health. Like I was literally going into psychosis a lot of times*,* but they will just turn me away. I’m like*,* ‘I really need to be in the hospital right now ’… Don’t turn them away. They could actually be in danger of harming themselves or others (Participant 40*,* Woman).*

*I would probably add a lot more counselors. I just want to stress that we need more mental health services. Because even the doctors and nurses will tell you*,* that [psychological supports] is what keeps people alive. It’s not the medications*,* always it’s the frame of mind. And if you get people that have history*,* that have all this other baggage*,* and they can get rid of some of that baggage*,* they [will] have more self-confidence. It’s easier to deal with the other crap [HIV]*,* with the medications and it becomes manageable. It’s easier to deal with that [HIV] if you’ve got the baggage dealt with (Participant 54*,* Woman).*

One woman participant advocated for more counselling targeted toward the unique needs of women who have experienced violence, as there remains a lack of resources/services addressing the effects of gendered forms of violence on people’s health and wellbeing.

*More one on one counseling for women*,* maybe more for girls. There is a lot of violence. I know what they’re going through*,* there are a lot of pigs [sexual assailants] out here (Participant 63*,* Woman).*

#### Peer support during HIV diagnosis

Participants of all genders recalled receiving a diagnosis as an extremely traumatic experience. Most said that they would have liked to talk to somebody who also lives with HIV to provide them with hope, guidance, and help them stay calm. Hearing about living with HIV in plain language and via informal conversations (rather than in a formal conversation with a health provider) was also perceived to be a useful strategy to ensure patients understand the nature of their diagnosis and feel comfortable to ask questions about what is going to happen to them. Many participants were on their own and with little social support at the time of their HIV diagnosis, and they would have appreciated a “role model” to show them it is possible to have a *life* after diagnosis.

*I think I was looking for someone to tell me that everything was going to be okay and what I needed to do was these steps to get where I’m trying to go and not give up (Participant 39*,* Woman).*

*I say it’s so important to [have] a peer there. So that they can say to them*,* ‘listen I’ve had it for 20 years*,* and I’m still here. It’s not a death sentence. You know*,* it’s a chronic illness that you have to deal with*,* period.’ Because it’s not a doctor. It’s not somebody who’s just clinical tech to tell you all the data and everything else. But if it’s somebody that’s taking the medications*,* lives it*,* deals with the side effects and all this. There’s a lot of little tricks in dealing with it that most people don’t [know]. It’s like*,* in those days*,* you know*,* when you were told you’re HIV positive*,* you didn’t plan ahead. And if I knew that I’d be alive today then*,* my life would have been completely different (Participant 48*,* Man).*

*Just knowing that there’s someone there sharing something that you have in common*,* that commonality is key*,* it can make a difference. Like whether you want to belong to something or not or feeling like you belong. Or*,* feeling like you are worth (Participant 57*,* Non-Binary).*

#### Social programming

Participants wanted more low-barrier, drop-in, and informal opportunities for connections with peers living with HIV. They highlighted the need for more access to peer resources and spaces to reduce internalized stigma, gain hope to live a fulfilling life with HIV, build self-confidence, support their mental health, and teach and learn unspoken realities of living with HIV.

*Confidence*,* it offers them. To learn about self respect. And the most important thing is that you learn about other people’s experiences*,* and you get strength from other people. For example*,* I knew there were people out there that were 5–10−20 years HIV positive*,* so I knew it was attainable for myself. And I know when I was molested when I was younger*,* and I had gone to some groups*,* when I could find them sporadically. I knew that there’s people surviving and thriving and I knew that would help me. And you learn things*,* you learn how people cope with certain situations*,* because it’s very similar. And you identify with them*,* you have a camaraderie. And this is what a lot of these groups help the most. Even some of the staff have said for years*,* it’s not the medication that keeps you alive*,* it’s your mind. And they don’t [have many social groups]*,* that’s unfortunate (Participant 54*,* Man).*

Some participants who self-identified as women suggested more groups for young women and their families. They wanted spaces where women could navigate sharing their HIV diagnosis with their family members. One man lamented the closure of men-specific resources and advocated for more support groups for men who have been sexually assaulted as children, suggesting the need for services that address gendered-based challenges and needs.

### Biomedical supports

Participants recommended changes to the way the medical delivery of HIV care occurs in Manitoba. The sentiment for these changes surrounded the need to take healthcare outside of the traditional clinics and into the places people newly diagnosed with HIV often access, such as emergency shelters.

#### HIV outreach

Participants noted the need for a dedicated HIV outreach team that goes into the community to bring HIV services to people. Participants emphasized that many people, especially those struggling with houselessness and substance use, will benefit the most from this expansion of services as they have the most challenges attending traditional HIV clinics.

*[I] envision some of them [PLHIV not connected to HIV care] would feel more safe for the healthcare providers to go in their area. If*,* for example*,* to go even more where they are. Or because they don’t feel safe going in [an HIV clinic]. Where here I kind of envision having a community outreach thing. I’m trying to put myself in their shoes (Participant 33*,* Man).*

*You got to have more outreach going to these people and saying hey how are you guys doing? Yeah if we come here [HIV clinic] it’s really good they take our blood and everything but it still not outreach*,* you gotta reach out to them… Seriously in all my fucking years of this [living with HIV]*,* I’ve never seen any outreach man with all the problems we have here … Nobody actually confronts a person and says*,* hey it seems like you are not doing well. And sometimes man*,* that’s just all fucking someone needs to hear*,* somebody they care about and [treats them] like a human instead of a fucking junkie (Participant 52*,* Man).*

*Outreach is part of the same thing. It’s [about] getting out*,* meeting people where they’re at*,* talking to them*,* like the tent cities*,* and being able to go in and like “Hi*,* I’m [name] and this is why I’m here. And if you want a [HIV] kit here they are. You want to see how they work. I can show you how they work”. I think we were to get a lot more people earlier in their diagnosis if we were able to do that (Participant 44*,* Man).*

#### HIV services outside 9 am– 5 Pm

Some participants discussed how traditional HIV clinic hours, Monday to Friday 9 am to 5 pm, create access barriers for many PLHIV, especially those who are using substances, as they tend to lose track of time. While they recognized the incredible work and how overworked and burnt-out HIV service providers are, they emphasized that expanding the hours of operations to weekends or after hours could better support PLHIV. Likewise, people recommended more availability for walk-in appointments as some people can forget their appointments when using substances.

*Oh*,* they’re going to lynch me for saying this. Saturday*,* be open Saturdays. I know nobody wants to work on a weekend. Sometimes it’s just for getting tested … Say you are a drug addict. I’m like*,* how about if I see you on a Saturday*,* rather than party (Participant 51*,* Man).*

#### Specialized care outside metropolitan areas

Participants living in rural and remote communities wanted more HIV services outside of Manitoba’s major cities, Winnipeg and Brandon. They noted the current lack of public provincial transportation and, with harsh winters, ground or air transportation can be very unreliable or even dangerous, making it difficult for residents from more rural or remote areas to attend care.

*Because coming here [HIV clinic]*,* for example it’s wintertime*,* it’s not that easy. Well*,* especially with that visibility (Participant 35*,* Man).*

*Have an HIV clinic on the reserve*,* or more services for HIV in the reserve … Oh*,* yes. I was flying in and out to get here for appointments. Depending on the weather or not*,* I could come by vehicle (Participant 49*,* Man).*

#### Universal coverage for HIV medicine

Participants advocated for HIV medicines to be fully accessible to everyone. They explained that the current provincial insurance plan is insufficient and almost pushes people to stop working so they can afford their coverage. In other words, when people are employed, they must pay high annual deductibles for their HIV medicines. These deductibles are, at times, too expensive and some people have ended their employment to receive provincial social assistance programs which do not require them to pay the deductible for medications.

*The big change that I would make is make the HIV meds free to everyone. Like not having to jump through these hoops*,* because they are so expensive*,* that even if somebody is working*,* and then gets HIV*,* they almost have to leave their job and go on welfare because the medication is so expensive. So*,* I’ve never been able to get my head around*,* why is it not free for everyone? (Participant 44*,* Man).*

### Social supports

Participants recommended social supports to provide wrap-around services that might assist PLHIV and respond to the multiple needs of PLHIV who are unhoused and/or struggling with addiction. The two major social support recommendations were (1) expanded transportation support to attend appointments and (2) emergency housing for PLHIV.

*Transportation. I think I’m just lucky to have a friend that had a car [during HIV diagnosis] that would take me to the hospital because if I would have taken a bus or walked I think it would took me forever. And I would have felt like all embarrassed or like if I might have needed an ambulance and then have an ambulance bill (Participant 39*,* Woman).*

*That I could use [transportation]*,* I don’t know a way to get there [HIV clinic]. That’s why I didn’t make the other ones [appointments] that’s why I couldn’t get here. That’s definitely a big one. I could definitely use more transportation (Participant 55*,* Woman).*

Participants also advocated for increased availability of emergency housing for people newly diagnosed to have a safe and stable place to focus on their HIV diagnosis and linkage to care. The complex realities of experiencing unstable housing relegate HIV care as people have to focus on other priorities to stay alive.

*Having the stability of my own place having a steady routine that’s how you get into not forgetting and take your pill every day. Because you have a home you go home that you can fell sleep. That you get up in you take your pill that is it (Participant 42*,* Non-Binary).*

*I’d say first one would be get better housing for us. So*,* at least have somewhere to go or just a place to crash. Because fucking shit can go wrong when you’re living on the streets*,* man. Yeah because fucking in the streets is easier to get sick too (Participant 61*,* Man).*

## HIV educational strategy

We found a need for a multifaceted educational strategy to make information about HIV and STBBIs more accessible across our data. Participants recounted many instances of internalized and enacted stigma and discrimination from primary healthcare providers and social circles. They mentioned that an educational strategy would not only provide precise information about symptoms to look for and places to go, but it would also destigmatize HIV by informing the general public and primary healthcare providers.

### Printed posters and billboards

Participants shared that many people at risk of acquiring HIV or those who are unsure if they already have HIV cannot get clear information, as many lack internet access. They depend on word of mouth, which may lead to misinformation about transmission, testing, and treatment options. To address this, participants suggested printed posters and billboards in areas where social services are concentrated, such as in downtown Winnipeg. There were different recommendations on what content to include, but most participants agreed that people need to know about symptoms to look for and places to go if they think they have HIV or an STBBI. Similarly, these materials should include friendly clear messages and avoid stigmatizing words that might deter people from seeking services. To help destigmatize HIV in the general population, participants suggested posters and billboards with information about U = U (Undetectable = Untransmissible). In this point, participants emphasized that the general population is not aware that once someone’s viral load reaches an undetectable level, they cannot transmit HIV. Participants described that mobilizing this message would shape HIV as a chronic illness rather than a ‘death sentence’.

*Make a pamphlet and put it up a bulletin board. Like with pictures and just writing like visual thing. Pills*,* probably bloodwork. I found those work. Maybe one with a nurse of where to get bloodwork and help for HIV and CD4s count and everything. Probably I would put them in the downtown (Participant 49*,* Man).*

*More pamphlets I guess more commercials billboards. Instead of just like posting them just in medical areas*,* like clinics and stuff like that*,* because I see a lot of posters in like clinics and stuff. Get more posters*,* like out there to people who will take them (Participant 40*,* Woman).*

*There’s a lot of people that go by and ride the bus on a daily*,* and a lot of people don’t know the numbers of how much of an epidemic it is in the city of HIV. But they’re scared of it and they don’t need to be scared of it … Nothing. It’s almost like they don’t want to talk about it. It’s almost like*,* the medical profession or the government doesn’t want to talk about it (Participant 59*,* Man).*

### Community meetings

To increase opportunities for peer connections, participants suggested more ‘open mic’ style meetings in community organizations. The suggestions for content of these meetings varied as some participants wanted more structured educational sessions to learn about HIV and STBBI. Others wanted meetings where they could ask about anything related to these topics without a particular structure. People emphasized that meetings would clarify HIV-related misinformation sometimes shared among community members, which fuels stigma and discrimination.

*I would put on these sex ed things every Friday. And it was two hours and you get pizza and 20 bucks. Just to learn about sex*,* and condoms (Participant 41*,* Woman).*

*Come up with like having one person who is living with HIV and sitting them in front and them explaining their journey like what happens when you didn’t want to take your medication. So*,* they can teach (Participant 53*,* Man).*

### Sex education in schools

A lack of comprehensive sex education in schools was noted by most participants, and they recommended that mandatory, comprehensive sex education become available in Manitoba. While some had received sex education in school, they felt the information provided was superficial (e.g., it would only show how to use an external condom) and never explained appropriate prevention or transmission of STBBIs.

*I thought Sex Ed was mandatory in Manitoba*,* I didn’t think we’re as bad as the States. But a 20-year-old thinking [they can] get it [HIV] from a toilet*,* that’s ridiculous. And the thing is*,* when they fall for somebody*,* and they don’t have that self confidence*,* they don’t even know how to approach talking about condoms (Participant 54*,* Man).*

### Training primary healthcare providers

Most participants reported stigma and discrimination from primary service providers. These participants emphasized the importance of updating mandatory training for primary healthcare providers to reduce stigma and discrimination, as many PLHIV were deterred from continuing care because of these experiences. Participants wanted providers to be more empathetic towards people burdened by poverty and houselessness.

*And we honestly need more Indigenous nurses*,* health care providers just to put themselves in their shoes. There’s so much stigma in the hospital. It’s just it’s very rarely [you] meet a lot of caring practitioners or doctors*,* it’s rare (Participant 40*,* Woman).*

*There’s training and when they’re become a nurse*,* but it’s so minimal. Well*,* even I had one nurse telling me that*,* you know*,* their book education [on HIV] was about an hour. I did these workshops at [University]*,* and I don’t know how many nurses came up to me and said*,* they figured out who I was*,* and all of a sudden*,* they’re all coming and visiting. And the most common comment was [that] they learned way more from me talking to them for 25 min*,* than they did from anything from that book (Participant 44*,* Man)*.

Two men strongly emphasized the need to train healthcare staff in prison settings. One of the men shared that the inhumane treatment he received while navigating an HIV diagnosis in prison made him consider never dealing with his HIV diagnosis.

*I would definitely change how the medical staff treat their patients. Like they are inmates yes*,* they did a crime yeah*,* maybe they didn’t do crime and you just didn’t know. But regardless*,* they’re still human. They’re not a fucking number. I would definitely change that about the penal system and people with illnesses like mine. Granted I’m doing my time yeah*,* I did something stupid*,* yes*,* and I am doing my time for it but I’m doing a little bit more fucking time than other guys because I’m fucking sick. I had to deal with this sickness*,* and I had to hide from everybody they should try to understand how that affects you. Just a little empathy man (Participant 52*,* Man).*

### Social media campaigns

Participants also suggested social media campaigns to reach younger people who may be at risk of acquiring HIV. Most participants agreed that younger generations will connect more through social media as they are already familiar with and using these platforms.

*Maybe through social media*,* when I feel it’s not talked about a lot. And everybody is. Most people are using social media*,* but you don’t really often see medical things talked about on social media (Participant 36*,* Man).*

## Discussion

This paper outlined the major recommendations for improving the HIV cascade of care in Manitoba from the perspective of PLHIV. ‘Meeting people where they are at’ encompasses strategies to mobilize HIV care beyond traditional medical facilities and into the places where people newly diagnosed with HIV, and especially those marginalized, spend their days. According to participants, this mobilization would bridge the hurdles that PLHIV must jump to receive HIV care, particularly for those experiencing houselessness and substance use disorders.

Participants suggested creating a dedicated HIV outreach team, expanding access to mental health services, increasing informal opportunities for peer connections and camaraderie, funding universal access to HIV medicine, housing, and transportation support, among other strategies to connect more PLHIV to on-going care. A recent systematic review and meta-analysis on the efficacy of HIV outreach teams on HIV care engagement in men showed promising results. In particular, outreach teams, including peers situated in non-clinical settings PLHIV frequent and trust, had higher HIV testing uptake, linkage to HIV care, and ART initiation among men [16]. Similarly, community-based HIV outreach prevention efforts across the world have proven beneficial in reducing HIV exposure behaviours and preventing HIV infection in underserved communities of people who inject drugs [17]. Evidence from the Seek and Treat for Optimal Prevention (STOP) of HIV/AIDS initiative based in British Columbia provides additional evidence for supporting HIV outreach models [18]. Employing a treatment as prevention strategy which included expanded HIV testing and immediate connection with free HIV medicines, researchers found a 64% shorter time from diagnosis to treatment initiation and a 21% shorter time from treatment initiation to viral suppression. Overall, the literature suggests that HIV outreach teams have greater success of preventing HIV and connecting PLHIV with HIV and substance use services. There is an established mobile public health service in Winnipeg which connects people around the city with harm reduction and health services [19], suggesting that given proper funding and resources this recommendation would be feasible in the larger Manitoba cities.

Men, women, and non-binary participants in this study wanted increased access to mental health services as the current services are insufficient, and do not consider gender-differences. As reported in the results, men and women suggested the need for services that address gendered-based challenges and needs. It is imperative for services to consider specific gendered experiences as they can shape how people shape their experiences with HIV prevention and care delivery [20–22]. Similarly, the literature suggest that expanded mental health services in HIV clinics could reduce detectable viral loads and improve rates of HIV viral suppression [23]. Participants also called for efforts to improved access for substance use treatments which have been found to reduce risk of HIV acquisition, particularly among people experiencing houselessness [24]. These findings are relevant to the Manitoba context as people newly diagnosed with HIV are experiencing increased mental health conditions, substance use disorders, and houselessness [2].

Participants also advocated for more opportunities to engage with a community of peers also living with HIV when receiving a diagnosis, and while maintaining regular HIV care. A recent study showed moderate to high effectiveness of improving HIV indicators such as retention in HIV care, improved adherence to HIV medicines, and better viral suppression levels in interventions that included direct face-to-face peer support compared to regular care [25]. Many participants in this study remarked on how these peer initiatives used to be available in Winnipeg but have slowly stopped. Given the rising cases of HIV in Manitoba, funding should be prioritized for such groups that foster peer interactions to support PLHIV in their HIV journey better.

A key recommendation from participants was expanding universal access for HIV medicines (e.g., antiretroviral therapy) so anyone could afford the medication without depending on third-party employer insurance or provincial insurance plans. Until 2023, Manitoba was one of the only provinces in Canada without universal coverage for HIV medicines [26]. Participants in our study explained that there are misconceptions about the actual costs of HIV medicine and some people believe they would have to pay for all HIV medicines out-of-pocket, which deters them from HIV care. Other participants spoke of increased anxiety and uncertainty because of having to pay high yearly deductibles, pushing some to stop working in order to be eligible for provincial insurance plans. Policy-makers should prioritize universal coverage as a cost-effective alternative in treating HIV given the associated costs of PLHIV who are not on HIV medicines. In 2008, the estimated net value of economic loss attributed to people diagnosed with HIV was $1.3 million per person [27], yet researchers in British Columbia have found that costs associated with PLHIV not on treatment are 3.4 times greater than those achieving viral suppression [28].

In addition, participants recommended a multifaceted educational strategy to improve awareness and reduce the stigma of HIV. The first part of the strategy included a media campaign using printed posters and billboards around places where people congregate to inform them about HIV services and reduce stigma towards PLHIV. Mass media campaigns have proven beneficial in preventing HIV through increased testing and safer sex education [29]. While posters have been shown to increase clinic attendance in HIV primary care clinics [30], less is known about their value in the places PLHIV in this study recommended, such as shelters and bus stops. Nevertheless, posters informed by PLHIV who have lived experiences of houselessness, and substance use disorders might provide an engaging mean for PLHIV to reconnect with their HIV care.

Second, participants in this study further advocated for more local events designed to inform community members about HIV. There is strong evidence that peer-led education can promote HIV testing and use of condoms and reduce sharing drug equipment and unprotected sex [31]. These findings support the design of community meetings with peers sharing information and stories as described by participants in this study. Thirdly, study participants recommended comprehensive sex education in schools. A systematic review of the effect of school-based sex education across thirty years provides strong support for establishing these programs [32]. The authors argue that these programs create opportunities for sexual diversity, recognizing signs of intimate partner violence and child sexual abuse, and developing healthy relationships [32]. The majority of participants in this study emphasized that the sex education they received was inadequate and needs an overhaul, especially considering the rising cases of HIV and STBBI in the province. In addition, participants suggested dedicated educational sessions for primary care providers on how stigma and discrimination keep PLHIV from getting the care they need as, participants in this study explained how they have been discriminated against in health spaces and deterred them from engaging with healthcare. Other research has also found that stigma and discrimination from healthcare providers is linked with lower HIV care utilization [33]. Lastly, social media interventions to improve HIV prevention, testing, linkage, and retention have shown to be beneficial in changing health behaviours and reaching a broader audience through low-cost options (e.g., Facebook) [34]. Overall, the educational strategies identified by participants are supported by evidence and could be low-cost strategies to inform people about HIV and reduce stigma towards PLHIV.

Canada has made tremendous progress to decrease the HIV incidence and improve treatment outcomes. However, Manitoba has seen an unprecedented increase in the HIV incidence, and reported a changing epidemiology of people newly diagnosed with HIV (similar rates of HIV between males and females, significant proportion of injection drug use, mostly methamphetamine, houselessness, mental health conditions and other social and structural barriers to HIV prevention strategies and HIV care). This changing epidemiology has been described in other high-income countries where these intersecting conditions are threatening the achievement of the UNAIDS 95-95−95 goals. Therefore, it is essential to center and incorporate people’s needs and priorities to improve access to HIV prevention and engagement in HIV cascade of care. It is our hope that these initiatives can take place in Manitoba and places with people living with HIV with similar sociodemographic characteristics to improve HIV prevention and care for everyone. We have used the findings here to support other stakeholders across the province in advocating for better services. Since the study took place, there have been many announcements providing increased funding opportunities and expansion of services that align with some of the recommendations that participants in this study described. For instance, the government of Manitoba announced on December 1st, 2023 and within the Budget 2024 an increase in funding for the Manitoba HIV program to support the infrastructure needed to provide more outreach services and case management and better connect people living with HIV who may be out care [35]. Similarly, there will be funding available for a mobile care service run by a trusted community-health organization that could provide HIV and STBBI care to people experiencing houselessness [35]. These changes resonate with the recommendations described throughout our theme of ‘meeting people where they are at.’ Similarly, in our study participants described how challenges for paying HIV-specific medication could deter some people from HIV care. On May of 2024, the government of Manitoba announced The Manitoba HIV Medications Program which provides full coverage to all HIV medications free of charge for those who did not have already established federal or provincial coverage [36]. Participants in our study described many changes that are needed for the improvement of HIV care in Manitoba, yet the announcements for expanded funding and services mark the start of province-wide changes that could better connect people with HIV care. Future research should elucidate the extent to which these new services have addressed the service gaps we have described in this paper or if there are new changes needed to better meet the needs of PLHIV.

This study has several limitations. First, our data collection occurred at HIV clinics, and we may not have reached PLHIV who have been out of care for a long time and/or who have never been connected to care. To minimize this, we engaged in intensive community consultations, guidance, and flexible and low-barrier recruitment strategies to reach people in and out of care, and those facing structural disadvantages. Second, the HIV clinics where we conducted data collection are located in the major metropolitan areas of Manitoba, which may underrepresent the views of people living in rural and remote communities. Nevertheless, we connected with our community partners to support recruiting PLHIV residents outside of these cities, which informed our findings. Third, qualitative studies by nature have smaller sample sizes than quantitative research or studies using population data, and this can limit their generalizability. Nevertheless, the richness of information provided gives in-depth understanding of how changes can be made to improve HIV care in Manitoba, which may be adaptable in other settings that have similar conditions.

## Conclusions

Without meaningful policy or system changes, Manitoba will continue to report increased HIV diagnoses, particularly among those experienced most structural disadvantages. To better respond to the changing realities of those newly diagnosed with HIV, HIV prevention and HIV cascade of care, Manitoba must prioritize the knowledge and lived experiences of PLHIV. This study provides clear person-centred strategies suggested by people living with HIV that could bridge the barriers faced by PLHIV when accessing and remaining in HIV care and expanding education and prevention about HIV. These measures could support Manitoba’s strategies in reaching the UNAIDS 95-95−95 goals [37].

## Data Availability

Individual qualitative participant data will not be available since it contains potential identifiable information. Data dictionaries, qualitative codebooks, and quantitative data will be available upon request on a case-by-case basis.
